# Effect of Vibration Pretreatment–Microwave Curing Process Parameters on the Mechanical Performance of Resin-Based Composites

**DOI:** 10.3390/polym16172518

**Published:** 2024-09-04

**Authors:** Dechao Zhang, Lihua Zhan, Bolin Ma, Jinzhan Guo, Wentao Jin, Xin Hu, Shunming Yao, Guangming Dai

**Affiliations:** 1 Light Alloys Research Institute, Central South University, Changsha 410083, China; zhangdechao@csu.edu.cn (D.Z.); guruci@163.com (J.G.); jwt2242444825@163.com (W.J.); ladihu4@gmail.com (X.H.); 2 State Key Laboratory of Precision Manufacturing for Extreme Service Performance, Central South University, Changsha 410083, China; 3 College of Mechanical and Electrical Engineering, Central South University, Changsha 410083, China; yaoshunming@csu.edu.cn; 4 College of Mechanical and Energy Engineering, Zhejiang University of Science and Technology, Hangzhou 310023, China; daigm@zust.edu.cn

**Keywords:** vibration pretreatment, microwave curing, thermogravimetric analysis, void content, microscopic morphology, impact strength

## Abstract

The vibration pretreatment–microwave curing process can achieve high-quality molding under low-pressure conditions and is widely used in the curing of resin-based composites. This study investigated the effects of the vibration pretreatment process parameters on the void content and the fiber weight fraction of T700/TRE231; specifically, their influence on the interlaminar shear strength and impact strength of the composite. Initially, an orthogonal experimental design was employed with interlaminar shear strength as the optimization target, where vibration acceleration was determined as the primary factor and dwell time as the secondary factor. Concurrently, thermogravimetric analysis (TGA) was performed based on process parameters that corresponded to the extremum of interlaminar shear strength, revealing a 2.17% difference in fiber weight fraction among specimens with varying parameters, indicating a minimal effect of fiber weight fraction on mechanical properties. Optical digital microscope (ODM) analysis identified interlaminar large-size voids in specimens treated with vibration energy of 5 g and 15 g, while specimens subjected to a vibration energy of 10 g exhibited numerous small-sized voids within layers, suggesting that vibration acceleration influences void escape pathways. Finally, impact testing revealed the effect of the vibration pretreatment process parameters on the impact strength, implying a positive correlation between interlaminar shear strength and impact strength.

## 1. Introduction

Due to the advantages of high specific strength, high specific stiffness [1], fatigue resistance, and designable material mechanical properties, carbon-fiber-reinforced resin matrix composites are widely used in aerospace [2], marine [3,4], rail transportation [5,6], military [7,8], and medical fields [9,10]. However, composite components are extremely sensitive to interlaminar delamination performance and impact resistance in the actual service process [11]. The mechanical properties of components are closely related to the molding process. Therefore, it is necessary to identify the influence of the curing process parameters on their mechanical properties to guide the design of composite components, reduce costs, and ensure product quality. At present, the autoclave curing process is still the most important molding method for composites, but its high energy consumption, long curing cycle [12], uneven temperature field distribution inside the components, and hysteresis in temperature control limit the development of autoclave curing technology. Scholars have gradually shifted their focus to the research of out-of-autoclave curing and molding technologies, such as UV curing [13,14], electron beam curing [15,16], laser curing [17,18], and microwave curing [19,20].

However, the microwave curing process has attracted extensive attention from researchers and scholars due to its unique “in-volume” and “selective heating” characteristics [21]. In the existing research on the microwave curing technology of composites, the main focus is on comparing the mechanical properties of the specimen formed using microwave curing and conventional thermal curing processes (the study refers exclusively to air circulation heating). For example, concerning the interfacial properties of composites, Hang et al. [22] found that the microwave curing process improved the fiber/resin interfacial bonding properties, increasing shear strength and flexural strength by 11.5% and 6.6%, respectively. Similar conclusions were also drawn by Haider et al. [23]. In terms of impact strength, De Vergara et al. [24] used a drop hammer to test microwave-cured basalt fiber composites with an increase in maximum load and penetration threshold by 17.8% and 14.5%, respectively. Dasari et al. [25] also found that microwave-cured laminates exhibited a 6% increase in peak impact force and an 18% increase in rebound energy. Chen et al. [26] compared the Charpy impact strength between microwave-cured and conventional thermal-cured specimens, concluding that microwave curing offered productivity advantages. However, both processes still had room for improvement in terms of the mechanical properties, affected mainly by void content. Nevertheless, microwave curing generally demonstrated superior mechanical properties compared to those under the conventional thermal curing process.

Therefore, in order to reduce the void content during the curing process of composites, research scholars have introduced the vibration energy into the curing process of composites. Muric-Nesic et al. [27] maintained a vibration frequency of 30 Hz for 30 min during the UV curing process of composites, and the void content was reduced to 0.3%. In order to explore the effects of vibrational forms, some studies have been conducted by introducing random vibrations during the curing process. Guan et al. [28] introduced random vibration into the microwave curing process, and the data showed that the experimental void content and mechanical properties were comparable to those achieved by the standard autoclave process. Yang et al. [29] also employed random vibration in the thermal curing process, and the average interfacial shear strength of the samples increased by 48.26% compared to the thermal curing process. However, the enhancement of mechanical properties was from the perspective of void content and the influence of fiber weight content due to changes in the process parameters not considered. For instance, the results of Xiang et al. [30] showed that the mechanical properties first increased and then decreased with the increase in fiber volume fraction. The same conclusion was obtained by Yang et al. [31], who conducted three-point bending tests on FeCrAl(f)/HA composite. Guo et al. [32] conducted a study focusing on the impact of short carbon fiber volume fraction on the impact resistance of composite, and the results showed that the impact resistance gradually increased as the fiber volume fraction increased from 0% to 11%. It is evident that fiber weight fraction has a significant effect on interlaminar shear and impact strengths. In conclusion, the recent studies have not fully explored the effect of vibration pretreatment–microwave process parameters on the fiber weight fraction of composites, nor have they adequately considered the combined effect of fiber weight fraction and void content on mechanical properties.

Therefore, this study employed orthogonal experimental design to investigate the effects of pretreatment temperature, dwell time, and vibration acceleration on the interlaminar shear strength of T700/TRE231 composite materials. TGA tests were conducted based on parameters corresponding to peak interlaminar shear strength, elucidating the influence of various fiber weight fractions on interlaminar shear strength. Concurrently, ODM was used to characterize the effects on the microstructure of voids. Finally, impact performance was evaluated using a drop hammer impact testing platform to assess the effects of the vibration pretreatment process parameters.

## 2. Materials and Methods

### 2.1. Materials and Equipment

The composite prepreg utilized in this study was a T700/TRE231, containing 58% fiber and 42% resin by volume, manufactured by Changzhou Tianqi Xinxin Technology Co., Ltd. in Changzhou, China. The prepreg has an areal density of 130 g/m^2^ and a single-layer thickness of 0.125 mm. The laminate size was 200 mm × 200 mm, with a plying orientation of [0/90/0/90/0/90/0/90]_s_.

**Figure 1:**
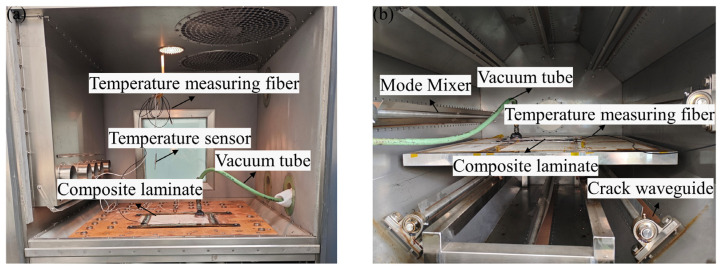
Vibration pretreatment–microwave curing equipment; (**a**) composite laminates in the vibration pretreatment equipment; (**b**) composite laminates in the microwave furnace.

The eighth layer of the composite laminate was embedded with a temperature measuring fiber to facilitate the subsequent monitoring and regulation of microwave curing temperature. The vibration pretreatment and microwave equipment were consistent with the literature [12], as shown in Figure 1. The laminate was placed into an octagonal microwave heating furnace for curing after vibration pretreatment.

### 2.2. Selection of Process Parameters and Curing Process Curve

Investigating the effects of the vibration pretreatment process parameters (vibration pretreatment temperature, dwell time, and vibration acceleration) on the interlaminar shear strength and impact performance of T700/TRE231, three levels were selected for each parameter. Vibration exhibits optimal void suppression when resin viscosity is at its lowest [12]. Therefore, for this study, pretreatment temperature parameters were chosen as 80 °C, 90 °C, and 100 °C, respectively. Based on prior research [28,29], the study range for the vibration acceleration parameters was between 5 and 15 g. This choice was influenced by the observation that when the vibration energy is too low, its void reduction effect is not significant, and when it is too high, it may lead to destructive outcomes. Hence, 5 g, 10 g, and 15 g acceleration parameters were selected. The process of void reduction took time, and a dwell time of 10, 30, and 50 min were selected, as depicted in Table 1.

**Figure 2:**
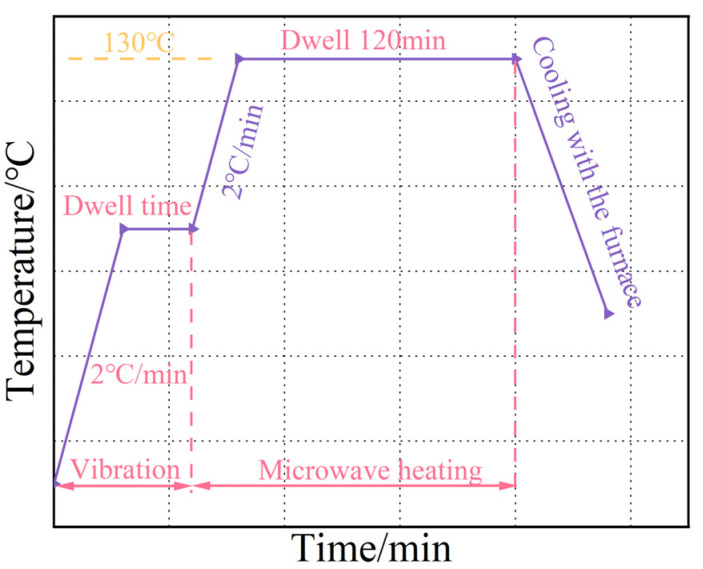
Vibration pretreatment–microwave curing process curve for T700/TRE231.

Figure 2 shows the vibration pretreatment–microwave curing process curve for the T700/TRE231 composite. The curve includes two parts: vibration and microwave heating. For the vibration pretreatment section, the laminate is heated up to the pretreatment temperature at a rate of 2 °C/min and maintained for a certain dwell time. The design parameters are shown in Table 1. The microwave heating section continues to heat at the same rate until reaching 130 °C and dwell time for 120 min. The curing process is carried out under the vacuum bagging pressure, followed by natural cooling in the furnace.

### 2.3. Interlaminar Shear Strength Test

**Figure 3:**
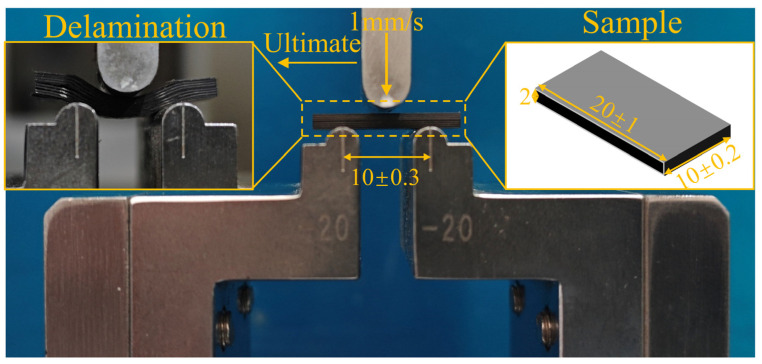
The testing process schematic and specimen dimensions of three-point bending.

To explore the impact of different process parameters on the interfacial strength of composite, the interlaminar shear strength was evaluated using the short beam three-point bending method. The testing process strictly followed the standards of the People’s Republic of China JC/T 773-2010 [33]. The testing process schematic and specimen dimensions were shown in Figure 3. The equipment used was the CMT5105 model produced by Sansi Taijie Electrical Equipment Co., China, in Zhuhai, Guangdong. When the indenter first contacted the surface of the specimen, a preload force of 0–30 N was maintained. Then, the indenter was lowered at a rate of 1 mm/min until the delamination failure of the sample occurred. The specimens were supported across a span of (10 ± 0.3) mm, with a bottom support radius of 2 mm and a punch radius of 5 mm. Four specimens were tested to determine the interlaminar shear strength of the process. The interlaminar shear strength was calculated using Equation (1):

(1) $$\tau_{In}=\frac{3F_{max}}{4bh}$$

where *τ_In_* represents interlaminar shear strength, and *F_max_* means the maximum load. *b* and *h* are the width and thickness of the specimen, respectively.

### 2.4. Characterization of Void Morphology

**Figure 4:**
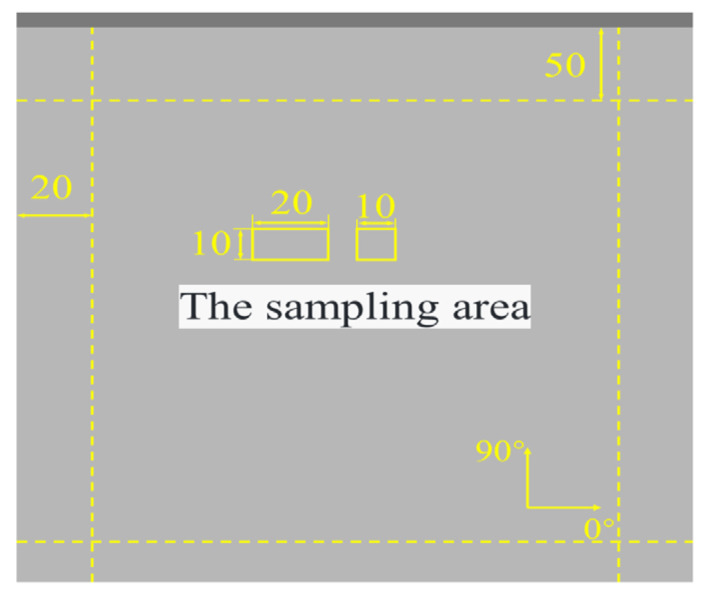
Sampling area for three-point bending and ODM specimens.

ODM was employed to investigate the void microstructure of samples subjected to vibration pretreatment–microwave curing processes. For each set of processing parameters, samples were collected from three distinct locations and averaged to ensure statistical accuracy. The samples, sized 10 × 10 mm, were shot in a 0° direction. Void analysis was conducted using Image Pro Plus 6.0 software, and void content was quantified according to Equation (2). The sampling area is illustrated in Figure 4.

(2) $$\gamma =\frac{S_{v}}{S_{a}}$$

where *γ* represents the void content of the specimen, and *S_v_* means the area of sample cross-sectional voids. *S_a_* is the area of the specimen cross-section.

### 2.5. Evaluation of Fiber Weight Fraction in Composite Laminates

**Figure 5:**
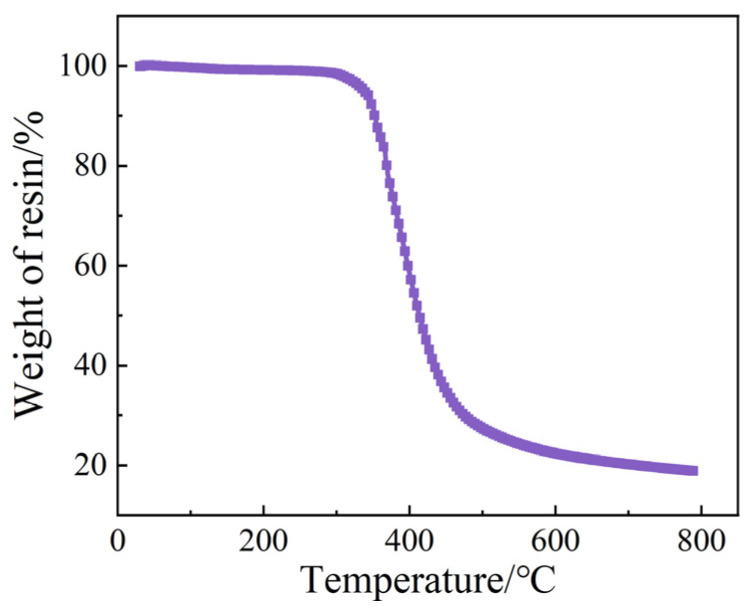
Variation in the weight of TRE231 resin with temperature.

To investigate the impact of vibration pretreatment parameters on the fiber weight fraction in laminates. The Platinum Elmer Corporate Management (Shanghai, China) Co., TGA/STA8000-FTIR-GCMS-ATD thermogravimetric-IR-gas chromatography analyzer was utilized. In order to make the experimental data more accurate, the samples were precisely cut, weighing approximately (20 ± 1) mg. The test conditions were as follows: the samples underwent controlled heating at a rate of 10 °C/min until reaching (785 ± 5) °C [34]; a continuous flow of nitrogen gas at a rate of 50 mL/min was maintained [35]. Figure 5 illustrates the variation in the weight of TRE231 resin with temperature. Resin decomposes rapidly at around (300–400) °C. At the end of the test, the residual resin percentage was 18.926%.

The fiber weight fraction of laminates processed could be accurately determined using Equation (3) [29,36]:

(3) $$C_{f}=(\frac{Res_{coms}-Res_{resin}}{1-Res_{resin}})\times 100\%$$

where *C_f_* represents the fiber weight fraction, and *Res_coms_* indicates composite residual weight fraction. *Res_resin_* is the resin residual weight fraction.

### 2.6. Testing Impact Properties of Composite Laminates

**Figure 6:**
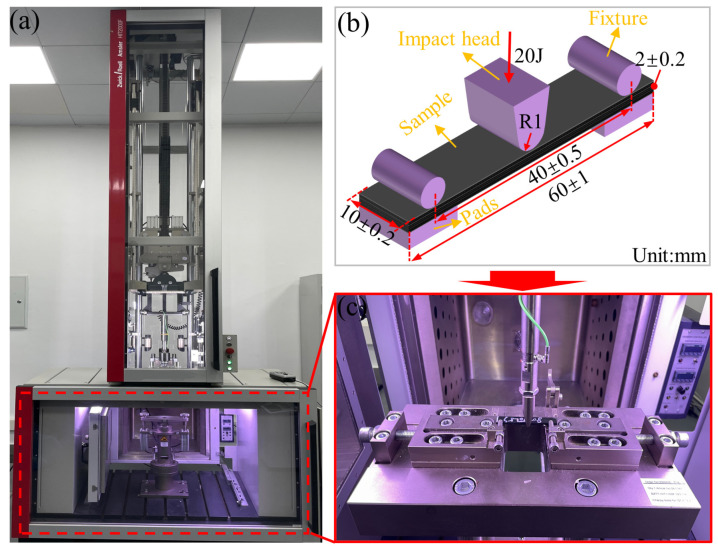
Drop hammer impact test equipment and impact schematic; (**a**) drop hammer impact equipment; (**b**) specimen dimensions and impact clamping; (**c**) simply supported beam impact process.

In various engineering fields such as aerospace, composite components need to withstand external loads at certain speeds, including collisions and impacts. Therefore, the impact performance of composite components was crucial for material design and selection. To investigate the effects of vibration pretreatment–microwave curing process parameters on the dynamic mechanical properties of composite laminates, a simply supported beam impact test method was employed. During the experiment, the relationship between impact force and time was recorded by sensors. The tests were conducted according to the standards of the People’s Republic of China GB/T 1043.1-2008 [37]. The HIT2000F device, manufactured by Zwick/Roell in Ulm, Germany, with a 9.294 kg hammer and a 2 mm diameter impactor was used, as shown in Figure 6a. The experiment was conducted at 25 °C with an impact energy of 20 J. The specimens were characterized for strength using notch-free through-the-thickness impact testing [38,39]. The specimen dimensions and span were (60 ± 2) mm × (10 ± 0.2) mm × (2 ± 0.2) mm (length × width × thickness) and (40 ± 0.5) mm, respectively, as shown in Figure 6b. Figure 6c illustrates the impact test process, and the calculation of simply supported beam impact strength for specimens was performed using Equation (4):

(4) $$I_{s}=\frac{E_{e}}{hb}\times 10^{3}$$

where *I_s_* represents impact strength (kJ/m^2^), and *E_e_* means energy absorbed in the destruction of the corrected specimen (J). *b* and *h* are the width and thickness of the specimen, respectively (mm).

## 3. Results and Discussion

### 3.1. Analysis of the Optimized Vibration Pretreatment Process Parameters

**Figure 7:**
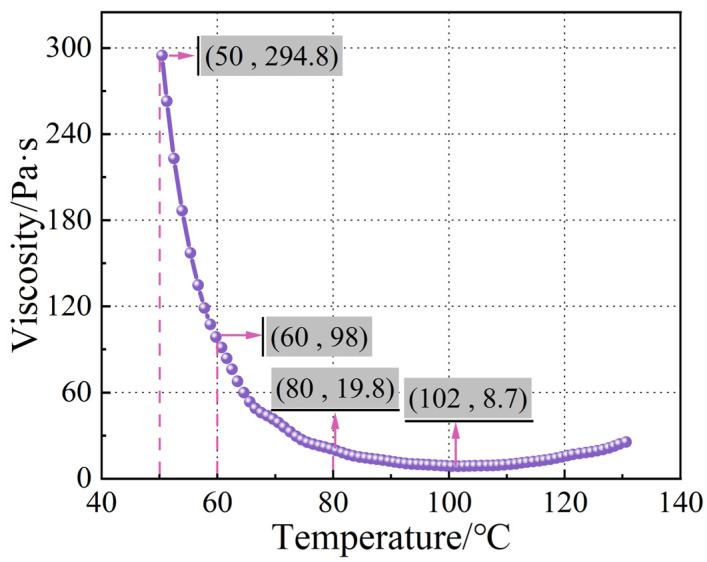
The temperature–viscosity curve of TRE231 resin.

As shown in Figure 7, the curve illustrates the variation in the viscosity of TRE231 resin with temperature. The test conditions for the curve were as follows: the temperature was increased from 50 °C to 130 °C at a heating rate of 2 °C/min, and the shear rate was 10 s^−1^. From the trend of the curve, the viscosity of the resin at 50 °C is 294.8 Pa·s, indicating a relatively high viscosity. As the temperature increases from 50 °C to 60 °C, the rate of viscosity change peaks and gradually decreases, reaching 98 Pa·s at 60 °C. The viscosity of the resin changes by 200.8% with just a 10 °C temperature difference, highlighting the high sensitivity of resin viscosity to temperature.

When the temperature rises to 80 °C, the resin viscosity decreases to 19.8 Pa·s. This can be attributed to the increased thermal motion of resin molecules with rising temperatures, leading to an increase in the average kinetic energy of the molecules and a weakening of intermolecular interactions. This enhances molecular mobility and facilitates easier movement through barriers between molecules, resulting in a reduction in resin viscosity. The viscosity of the resin changes slowly between 90 and 110 °C, reaching a minimum viscosity of 8.7 Pa·s at 102 °C. As the temperature continues to rise, molecular cross-linking reactions begin in the resin, gradually forming a three-dimensional network structure. The increase in molecular weight and complexity of the resin system limits the free radicals in the resin, leading to a subsequent increase in viscosity [40].

Table 2 shows the orthogonal experimental results of the interlaminar shear strength and void content for different vibration pretreatment process parameters. It can be seen that there is a negative correlation between void content and interlaminar shear strength [41], meaning that the higher the void content, the lower the interlaminar shear strength. The reason for the above phenomenon is that high void content reduces the actual contact area between materials. Additionally, voids induce stress concentration under applied forces. These factors collectively result in a significant reduction in interlaminar shear strength. It is evident that when the vibration acceleration is set at 10 g, the interlaminar shear strength consistently reaches its maximum value and has the lowest void content. Therefore, the vibration acceleration factor is initially identified as the primary factor influencing both interlaminar shear strength and void content, which is consistent with the previous research conclusion [12]. The order of dwell time and pretreatment temperature is opposite, and this discrepancy may be attributed to differences in resin systems. Based on the parameter analysis results in this study, the order of influence of vibration pretreatment process parameters on interlaminar shear strength is as follows: vibration acceleration > dwell time > pretreatment temperature. The optimized vibration pretreatment process parameters derived from the orthogonal experimental results indicate a microwave curing process with a pretreatment temperature of 90 °C, a dwell time of 30 min, and a vibration acceleration of 10 g (referred to as the 90 °C–30 min–10 g microwave curing process for brevity).

### 3.2. Effect of Vibration Pretreatment Process Parameters on Fiber Weight Fraction

**Figure 8:**
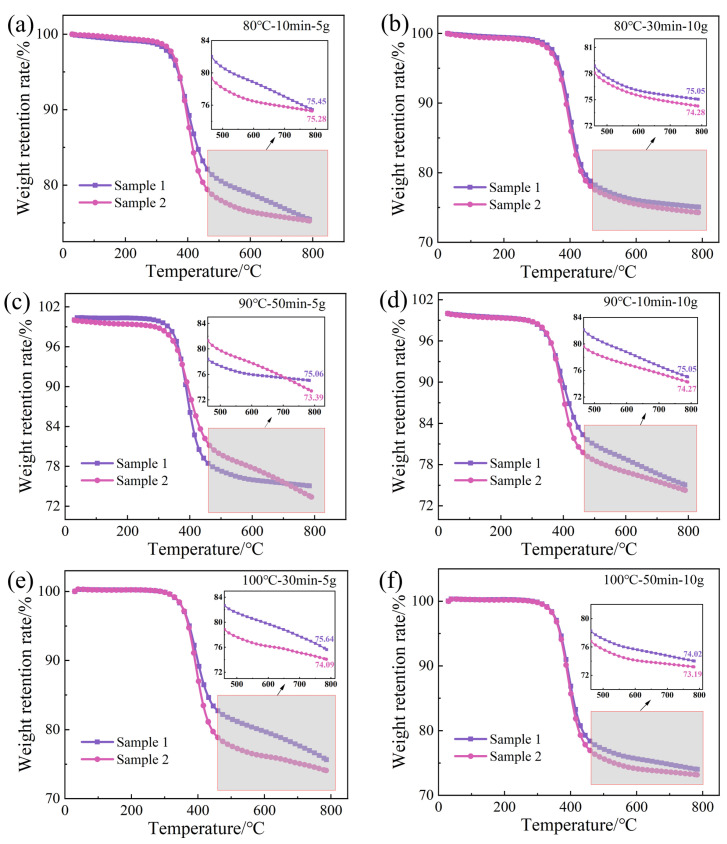
Weight retention rate of samples under vibration pretreatment parameters.

To elucidate the influence of vibration pretreatment process parameters on the fiber weight fraction in composite laminates, TGA was conducted on the pretreatment processes corresponding to the maximum and minimum interlaminar shear strengths at vibration pretreatment temperatures. Figure 8 illustrates the weight retention rate of composite specimens with temperature changes under different vibration pretreatment process parameters. At lower temperatures, the weight retention rate slightly decreases, mainly due to the conversion of moisture in the composite into water vapor [29] and expulsion as the temperature rises. When the temperature reaches 300–400 °C, the rate of change in weight retention rate gradually increases to a maximum and then decreases. At this stage, the resin begins to reach its optimal decomposition temperature, and the rate of resin decomposition gradually accelerates until it reaches a maximum. As the temperature continues to rise and with time, the resin content in the specimen rapidly decreases. It leads to a rapid overall decrease in weight retention rate. The remaining resin in the specimen completely decomposes, and the variation in weight retention rate eventually stabilizes. The values enclosed within the box in the figure represent the residual weight fraction of the sample following testing. This includes both the residual weight fraction of the resin (i.e., the carbon remaining after combustion) and the weight fraction of the fibers. 

**Figure 9:**
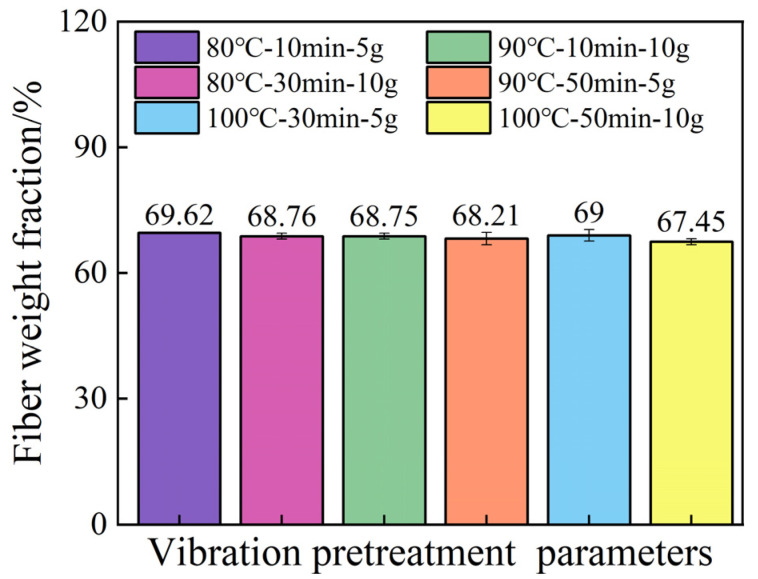
The fiber weight fraction of samples under vibiration pretreatment parameters.

The fiber weight fraction in the prepreg is 67%. According to Figure 9, the minimum fiber weight fraction under different vibration pretreatment process parameters is approximately 67.45%. This indicates that the composite laminates indeed undergo a compaction process during pretreatment. The TRE231 resin has a low viscosity in the range of 8.7 to 19.8 Pa·s at 80–100 °C. The introduction of a vibrational energy field results in a good impregnation effect, and excess resin has been squeezed out of the laminate. It can also be observed from the figure that within the range of pretreatment parameters study in this research, the fiber weight fraction has changed by 2.17%, suggesting that the variation in fiber weight fraction has a minimal impact on interlaminar shear strength.

### 3.3. Effect of Vibration Pretreatment Process Parameters on Void Micromorphology

**Figure 10:**
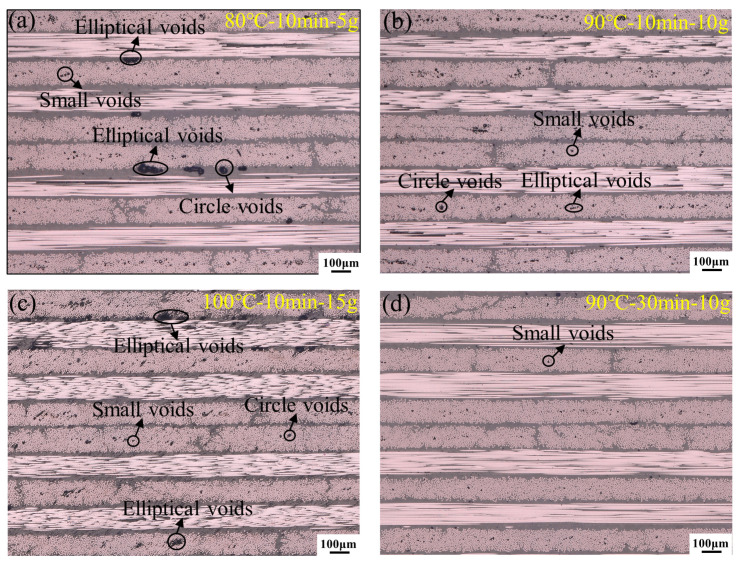
Microscopic morphology of samples under vibration pretreatment parameters.

Figure 10 depicts the microstructure of the specimen under different vibration pretreatment process parameters. According to Section 3.1, pretreatment temperature and dwell time have minimal impact on interlaminar shear strength. Therefore, only the microscopic morphology under variable acceleration and optimized pris examined. It is noticeable that when the vibration pretreatment parameters are set at 80 °C–10 min–5 g, there are numerous interlaminar elliptical and circular voids and a significant number of small-sized intralaminar voids. This situation is markedly different from the previous one, where voids were primarily distributed between the layers [12]. As the vibration energy increases to 10 g, interlaminar voids disappear, replaced by elliptical, circular, and small-sized intralaminar voids. When vibration energy is absent, the internal void pressure, hydrostatic pressure and vacuum bag pressure inside the laminate are in equilibrium, and the voids are distributed in the resin with a certain diameter. The introduction of vibration energy disrupts this equilibrium. According to Equation (5) [27], the alternating positive and negative forces induced by vibration cause large interlaminar voids to fracture into smaller voids under the influence of negative vibration forces, ultimately shifting from interlaminar to intralaminar spaces.

(5) $$P_{v}-P_{r}-P_{e}-P_{vibr}=\frac{K\sigma_{v}}{R_{v}}$$

where *P_v_* represents internal pressure of the void, and *P_r_* is hydrostatic pressure. *P_e_* is vacuum bag pressure, and *P_vibr_* represents pressure generated by vibration. *K* is the surface tension coefficient, and *σ_v_*, *R_v_* are void surface tension and radius.

The original small voids partially dissolve into the resin, as shown in Figure 10c. When the vibration energy increases to 15 g, interlaminar voids reappear, indicating that the magnitude of vibration energy can affect the escape path of voids. Figure 10d describes the void distribution and morphological features under the optimized vibration pretreatment process parameters. Compared to Figure 10a, large elliptical and circular voids have completely disappeared, and more small-sized intralaminar voids are observed.

### 3.4. Effect of Vibration Pretreatment Process Parameters on Impact Strength

**Figure 11:**
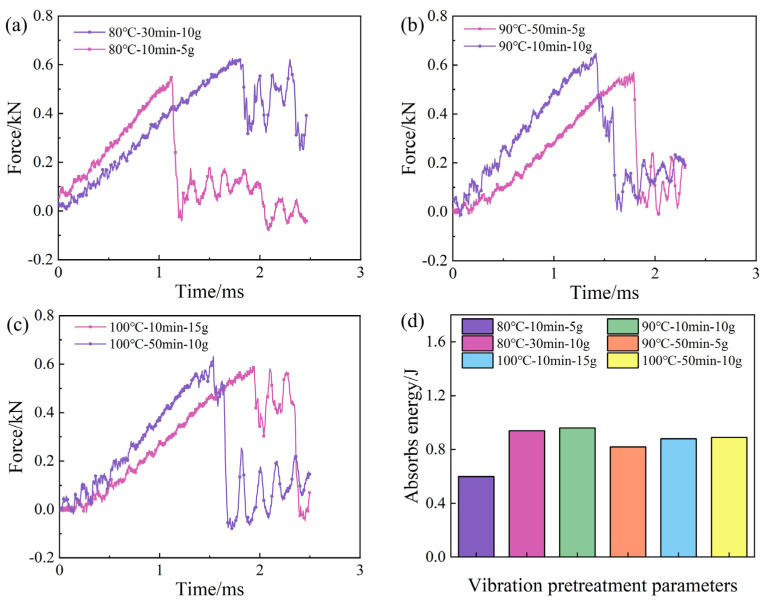
The impact time–force curves and impact strength of samples under pretreatment parameters.

Figure 11 illustrates the impact load–time curves and impact strengths of simply supported beams for specimens with different pretreatment process parameters. In Figure 11a–c, the variation of load with time exhibits oscillatory linear growth until reaching the maximum load [42]. This oscillation is mainly caused by the force generated when the impact head contacts the specimen. Additionally, since composites are linear elastic materials, the impact force gradually increases with time until reaching the maximum load. At this stage, the composite laminate remains undamaged and stays the elastic region. When the impact force exceeds the elastic limit of the composite material, fiber fracture, delamination, and matrix cracking start to occur [1]. The decrease in stiffness and the weakened load-bearing capacity of the composite laminate leads to a sudden drop in load. Subsequent oscillations may be caused by behaviors such as the energy absorption and crack propagation of the composite laminate under impact loading.

Figure 11d shows the impact energy absorption of simply supported beam specimens under different vibration pretreatment–microwave curing process parameters. It can be observed that the specimen absorbs the highest impact energy when the vibration acceleration is 10 g at the same pretreatment temperature. This is primarily influenced by the internal void content of the laminate. In terms of void content, when the vibration acceleration is 10 g, there is a lower void content in the laminate with almost no large voids visible compared to conditions at 5 g and 15 g. This indicates that the internal structure of the laminate is more compact, increasing the number of connection points and contact surfaces to effectively transmit and disperse impact energy, thereby enhancing the load-bearing capacity. Additionally, the low void content helps reduce the initiation points of cracks, shortening the path for energy absorption and dissipation, thereby slowing down the rate of crack propagation.

Table 3 presents the impact strength results calculated according to Equation (4), which indicate a positive correlation between impact strength and interlaminar shear strength. This correlation primarily arises because interlaminar shear strength denotes the bonding strength between the layers of composite laminates and their ability to resist external shear forces. When the interlaminar shear strength of composite laminates is high, it effectively prevents crack expansion and enhances the overall strength and toughness. Impact strength, on the other hand, refers to the material’s load-bearing capacity under external impact forces. Composite laminates with higher interlaminar shear strength are better at absorbing and dispersing impact energy, which slows down the damage process and increases their ability to withstand peak impact loads. Therefore, the higher the interlaminar shear strength in composite laminates, the more effectively they prevent crack propagation and enhance their impact resistance.

## 4. Conclusions

In this study, the influence of vibration pretreatment–microwave curing process parameters on the interlaminar shear strength of T700/TRE231 composite materials was firstly explored through an orthogonal experimental design. Based on the extremum of interlaminar shear strength, thermal analysis was conducted on samples corresponding to process parameters, clarifying the effect of fiber weight fraction on interlaminar shear strength. ODM was used to examine the void morphology of samples treated with different vibration pretreatment parameters. Finally, the impact performance of samples was evaluated through a simply supported beam drop hammer impact test. The main findings of this study are as follows:

(1) The order of pretreatment process parameters affecting the interlaminar shear strength of T700/TRE231 is as follows: vibration acceleration > dwell time > pretreatment temperature. The optimized parameters are a pretreatment temperature of 90 °C, a dwell time of 30 min, and a vibration acceleration of 10 g.
(2) Within the range of vibration pretreatment process parameters studied in this paper, the minimum fiber weight fraction increased by 0.45% over the initial prepreg, indicating that the pretreatment process produced a compaction effect. However, the maximum range of variation in fiber weight fraction is only 2.17%. Therefore, the influence of pretreatment process parameters on the fiber weight fraction is relatively slight.
(3) When the vibration energy is 5 g, large circular and elliptical voids are present in the samples. Increasing the vibration energy to 10 g caused large voids between layers to fracture into smaller voids and moved into within the layers. Further increasing the energy to 15 g reintroduces interlaminar voids, indicating that vibration acceleration can alter the escape path of voids.
(4) The simply supported beam impact tests of samples treated with different process parameters revealed that samples subjected to a vibration energy of 10 g exhibited higher impact strength, mainly attributed to lower void content. Additionally, the impact strength showed a positive correlation with interlaminar shear strength.

## Figures and Tables

**Table 1:** Orthogonal experimental design factors and levels (1 g = 9.8 m/s^2^).

	Factor	Pretreatment Temperature/°C	Dwell Time/min	Vibration Acceleration/g
Level				
I		80	10	5
II		90	30	10
III		100	50	15

**Table 2:** Orthogonal experimental results of L_9_(3^4^).

Test No.	Pretreatment Temperature/°C	Dwell Time/min	Vibration Acceleration/g	Interlaminar Strength/MPa	Void Content/%
1	80	10	5	48.52	0.98
2	80	30	10	58.18	0.57
3	80	50	15	53.89	0.71
4	90	10	10	59.44	0.44
5	90	30	15	55.82	0.66
6	90	50	5	48.60	0.80
7	100	10	15	52.23	0.77
8	100	30	5	51.65	0.78
9	100	50	10	55.58	0.67
Level Ι	53.53	53.40	49.59		
Level II	54.62	55.22	57.73		
Level III	53.15	52.69	53.98		
Extreme variance	1.47	2.53	8.14		

**Table 3:** Impact strength of different vibration pretreatment–microwave process parameters.

Pretreatment Temperature/°C	Dwell Time/min	Vibration Acceleration/g	Interlaminar Strength/MPa	Absorbs Energy/J	Impact Strength/(kJ/m^2^)
80	10	5	48.52	0.60	29.94
80	30	10	58.18	0.94	47.14
90	10	10	59.44	0.96	47.95
90	50	5	48.60	0.82	40.96
100	10	15	52.23	0.88	44.00
100	50	10	55.58	0.89	44.19

## Data Availability

The data that support the findings of this study are available from the corresponding author upon reasonable request.
